# Supplementary material for: Complex‐centric proteome profiling by SEC‐SWATH‐MS
Source: Mol Syst Biol. 2019 Jan 14;15(1):e8438. doi: 10.15252/msb.20188438 (PMC6346213; doi:10.15252/msb.20188438)
Supplement: Supplementary file 7 — Dataset EV6 [file MSB-15-e8438-s007.zip › feature_plots_bioplex/Q96P53.pdf]

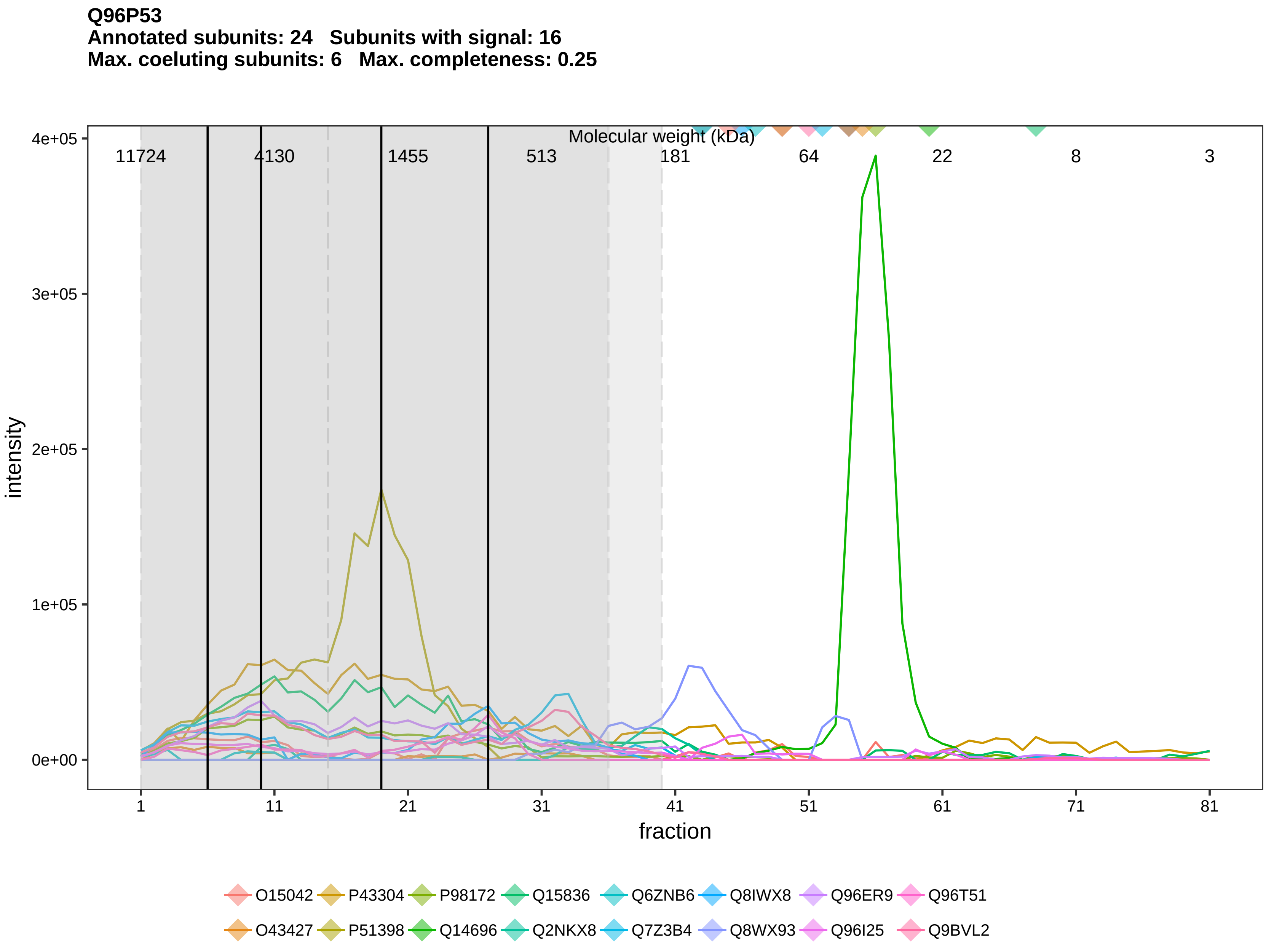
Q96P53
Annotated subunits: 24 Subunits with signal: 16
Max. coeluting subunits: 6 Max. completeness: 0.25
Molecular weight (kDa)
11724
4130
1455
513
181
64
22
8
3
4e+05
3e+05
2e+05
1e+05
0e+00
intensity
1
11
21
31
41
51
61
71
81
fraction
O15042
P43304
P98172
Q15836
Q6ZNB6
Q8IWX8
Q96ER9
Q96T51
O43427
P51398
Q14696
Q2NKX8
Q7Z3B4
Q8WX93
Q96I25
Q9BVL2
